# Dipeptide Transport Systems at the Interface of Peptide Metabolism and Drug Delivery in Cancer

**DOI:** 10.3390/ijms27093728

**Published:** 2026-04-22

**Authors:** Kyung-Hee Kim, Byong Chul Yoo

**Affiliations:** 1 Department of Applied Chemistry, School of Science and Technology, Kookmin University, Seoul 02707, Republic of Korea; kyungheekim@kookmin.ac.kr; 2 Antibody Research Institute, Kookmin University, Seoul 02707, Republic of Korea; 3 Diagnostic Research Team, InnoBation Bio R&D Center, Seoul 03929, Republic of Korea

**Keywords:** dipeptide transporters, PEPT1, PEPT2, SLC15 family, tumor metabolism, peptide transport, transporter-mediated drug delivery, tumor microenvironment

## Abstract

Protein turnover and extracellular proteolysis continuously generate diverse peptide fragments within biological systems, yet the metabolic and pharmacological implications of these peptides remain incompletely understood. Among these transporters, members of the solute carrier family 15 (SLC15), including peptide transporter 1 (PEPT1/SLC15A1) and peptide transporter 2 (PEPT2/SLC15A2), mediate the proton-coupled uptake of dipeptides, tripeptides, and structurally related compounds across cellular membranes. While these transporters have been extensively studied in the context of intestinal peptide absorption and drug delivery, their potential roles in cancer biology remain incompletely understood. Tumor microenvironments are characterized by extensive proteolysis and dynamic metabolic remodeling, processes that can generate diverse peptide fragments derived from extracellular matrix proteins and intracellular protein turnover. These peptides may accumulate locally and potentially serve as substrates for cellular peptide transport systems. Once internalized through peptide transporters, dipeptides are typically hydrolyzed into free amino acids that can support biosynthetic pathways, energy metabolism, and cellular growth. In addition to their potential metabolic roles, certain endogenous dipeptides have also been reported to influence cellular signaling pathways and redox homeostasis. The broad substrate specificity of peptide transporters has also attracted significant interest in pharmacology because numerous clinically used drugs exploit these transport systems for efficient cellular uptake. This property raises the possibility that peptide transporters may be utilized for transporter-mediated drug delivery strategies, including the development of peptide-modified prodrugs or dipeptide–drug conjugates. In this review, we summarize the molecular characteristics and physiological functions of dipeptide transport systems with a particular focus on the SLC15 transporter family. We then discuss emerging evidence linking peptide transporters to tumor metabolism and the tumor microenvironment. Finally, we highlight current progress and future perspectives in exploiting peptide transport systems for transporter-mediated drug delivery and therapeutic targeting in cancer.

## 1. Introduction

Peptides derived from protein digestion and intracellular proteolysis represent an important class of small biomolecules involved in nutrient transport, metabolic regulation, and intercellular communication. Among these, dipeptides and tripeptides are widely present in biological systems and can be transported across cellular membranes through specialized peptide transport systems. The major transporters responsible for the uptake of small peptides belong to the solute carrier family 15 (SLC15), which includes peptide transporter 1 (PEPT1/SLC15A1) and peptide transporter 2 (PEPT2/SLC15A2) [1,2,3,4]. These proton-coupled transporters mediate the cellular uptake of a broad range of di- and tripeptides as well as numerous peptide-mimetic compounds [5,6].

Dipeptide transporters were initially characterized in the context of intestinal nutrient absorption and renal peptide reabsorption, where they play essential roles in the efficient uptake and recycling of dietary peptides [3,7]. PEPT1 is predominantly expressed in the small intestine and functions as a high-capacity transporter responsible for the absorption of dietary oligopeptides, whereas PEPT2 exhibits higher substrate affinity and is mainly expressed in the kidney and several extraintestinal tissues, contributing to peptide reuptake and systemic peptide homeostasis [5,8,9]. Importantly, the substrate recognition of these transporters is remarkably promiscuous, allowing them to transport structurally diverse peptides and peptide-like molecules [10,11,12]. This broad specificity has attracted considerable interest in pharmacology because several clinically used drugs, including β-lactam antibiotics and antiviral prodrugs, utilize peptide transport systems to facilitate cellular uptake [12,13,14,15].

**Figure 1:**
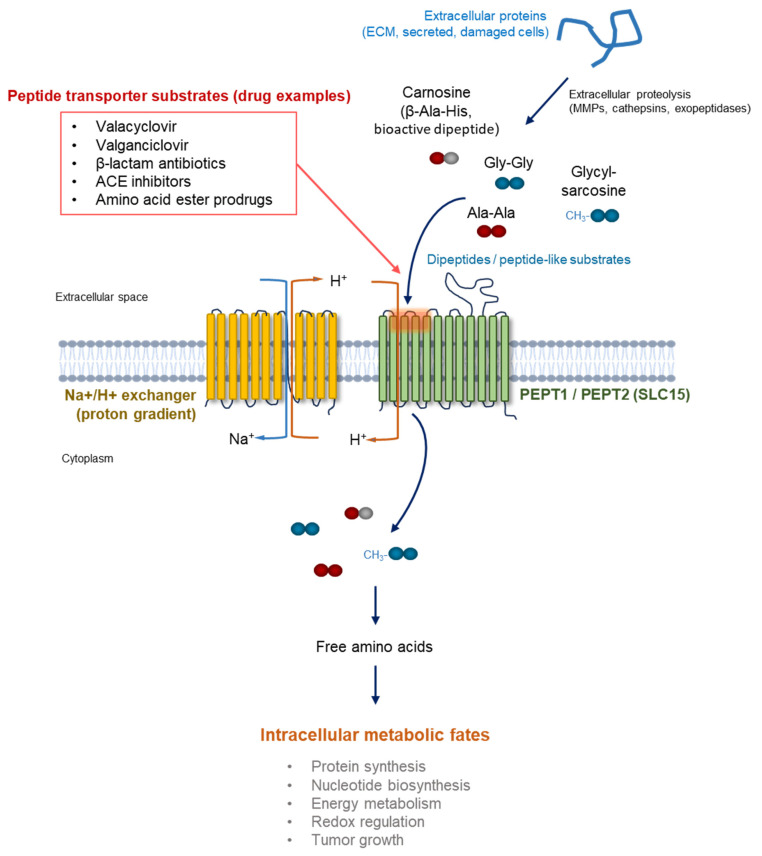
Conceptual framework linking extracellular proteolysis, dipeptide transport, and metabolic or pharmacological outcomes in tumor microenvironments. Extracellular proteins derived from the extracellular matrix, secreted proteins, or damaged cells can undergo proteolytic degradation within tumor microenvironments, generating peptide fragments including dipeptides. These peptides may accumulate locally and become available for uptake through peptide transporters such as PEPT1 and PEPT2. Once internalized, dipeptides are typically hydrolyzed to release amino acids that contribute to cellular metabolic pathways. In addition to serving as potential metabolic intermediates, certain dipeptides may influence cellular metabolic regulation. Furthermore, peptide transport systems can facilitate the uptake of peptide-mimetic drugs and prodrugs, providing opportunities for transporter-mediated drug delivery strategies.

Beyond their classical roles in nutrient absorption and drug pharmacokinetics, peptide transport systems may also represent an interface between protein degradation and cellular metabolism. Proteolytic processes occurring during dietary digestion, intracellular protein turnover, and extracellular matrix remodeling generate a diverse pool of peptide fragments that can serve as potential substrates for peptide transporters (Figure 1). These peptides may subsequently contribute to intracellular amino acid pools following transport and hydrolysis, thereby linking protein degradation to downstream metabolic pathways [5,6]. In this context, dipeptides may represent an intermediate metabolic reservoir connecting proteolytic processes with cellular biosynthesis and energy metabolism.

Cells undergoing metabolic stress frequently activate adaptive nutrient acquisition strategies to maintain metabolic homeostasis. For example, rapidly proliferating cells can increase the uptake of glucose, amino acids, and other nutrients through coordinated regulation of membrane transport systems [16,17,18,19]. In addition to classical nutrient uptake pathways, alternative mechanisms such as macropinocytosis, protein scavenging, and lysosomal protein degradation can provide additional sources of metabolic substrates [20,21,22,23]. These processes may generate peptide intermediates that could potentially be transported into cells through peptide transport systems.

Recent studies have reported altered expression of peptide transporters in several pathological contexts, including certain cancers, particularly in gastrointestinal and hepatobiliary malignancies [24,25,26]. In parallel, advances in metabolomics and peptidomics have revealed that circulating dipeptides are detectable in biological fluids and tissues, suggesting that small peptides may represent previously underappreciated components of systemic metabolism [27,28,29]. Although the quantitative contribution of peptide-derived nutrients to cellular metabolism remains unclear, these observations raise the possibility that peptide transport may participate in broader metabolic networks.

In addition to potential metabolic roles, peptide transporters have attracted interest as pharmacological gateways for drug delivery. Because PEPT1 and PEPT2 recognize general structural features of peptide bonds rather than specific amino acid sequences, they can transport a wide variety of peptide-like therapeutic compounds [5,30,31]. The successful development of several peptide-mimetic drugs exploiting peptide transport systems has demonstrated the feasibility of transporter-mediated drug delivery strategies [32,33]. These examples highlight how peptide transport pathways can be harnessed to enhance drug absorption, tissue distribution, and intracellular delivery [34,35].

Although the contribution of peptide-derived nutrients to tumor metabolism remains less clearly defined than that of glucose or free amino acids, the presence of diverse peptide fragments within tumor microenvironments suggests that peptide metabolism may represent an additional layer of metabolic complexity. Tumor tissues are characterized by extensive extracellular proteolysis and dynamic protein turnover, processes that can generate peptide intermediates that may become available for cellular uptake or metabolic processing [16,17,18,19]. While it remains uncertain to what extent dipeptide uptake directly contributes to tumor growth, peptide transport systems may nevertheless represent an interface linking extracellular protein degradation, peptide metabolism, and transporter-mediated uptake of peptide-like compounds.

In this review, we propose that dipeptide transport systems may represent an incompletely understood interface linking peptide metabolism, tumor microenvironmental proteolysis, and transporter-mediated drug targeting in cancer. We summarize the molecular characteristics and physiological functions of SLC15 transporters and discuss emerging evidence linking peptide transport systems to tumor metabolism. We further highlight how these transport pathways may be exploited for transporter-mediated drug delivery strategies in cancer.

## 2. Molecular Characteristics of the SLC15 Peptide Transporter Family

Dipeptide transport across cellular membranes is primarily mediated by members of the solute carrier family 15 (SLC15), a group of proton-coupled oligopeptide transporters responsible for the uptake of small peptides and peptide-like molecules [4,5,6]. The SLC15 family comprises four known transporters in mammals: PEPT1 (*SLC15A1*), PEPT2 (*SLC15A2*), PHT1 (*SLC15A4*), and PHT2 (*SLC15A3*) (Table 1). Among these, PEPT1 and PEPT2 are the most extensively studied and represent the principal transporters responsible for the uptake of dipeptides and tripeptides across plasma membranes [3,5,7].

Both PEPT1 and PEPT2 belong to the major facilitator superfamily (MFS) of membrane transporters and share a conserved structural organization characterized by twelve transmembrane α-helices forming a central substrate-binding cavity [10,37,38,39]. These transporters operate through a proton-coupled symport mechanism, in which peptide transport is driven by the transmembrane proton gradient [4,6]. In this process, extracellular protons bind to the transporter and facilitate conformational changes that enable the co-transport of peptide substrates into the cytoplasm [37,38]. This proton-coupled transport mechanism allows cells to efficiently accumulate peptide substrates even when extracellular peptide concentrations are relatively low.

Despite their structural similarity, PEPT1 and PEPT2 exhibit distinct functional properties. PEPT1 functions as a low-affinity, high-capacity transporter, enabling efficient uptake of dietary peptides in the small intestine where peptide concentrations are relatively high [3,7]. In contrast, PEPT2 operates as a high-affinity, low-capacity transporter, allowing efficient reabsorption of peptides in tissues where peptide concentrations are lower, such as the kidney and certain extraintestinal tissues [8,9]. These complementary kinetic properties enable coordinated peptide transport and systemic peptide homeostasis across different physiological environments.

One of the most distinctive features of dipeptide transporters is their remarkably broad substrate specificity. Rather than recognizing specific amino acid sequences, PEPT1 and PEPT2 primarily recognize the peptide backbone structure, including the presence of an amide bond and the spatial arrangement of amino acid side chains [10,11]. As a result, these transporters are capable of transporting an extensive range of substrates, including thousands of naturally occurring dipeptides and tripeptides as well as numerous peptide-mimetic compounds [5,12]. This structural promiscuity is particularly relevant for pharmacology, as many drugs that structurally resemble small peptides can utilize peptide transporters for cellular uptake.

The substrate spectrum of dipeptide transporters extends beyond natural peptides to include several classes of clinically important drugs. Notably, β-lactam antibiotics, angiotensin-converting enzyme (ACE) inhibitors, and antiviral prodrugs such as valacyclovir have been shown to exploit peptide transport systems for efficient absorption and distribution [12,13,14,15]. These findings highlight the pharmacological significance of dipeptide transporters and demonstrate their potential as targets for transporter-mediated drug delivery strategies.

Recent advances in structural biology have further improved our understanding of peptide transporter function. High-resolution structural studies of bacterial homologs and mammalian transporter models have revealed the alternating-access transport mechanism, in which the transporter alternates between outward-facing and inward-facing conformations to translocate peptide substrates across the membrane [37,38,40]. These studies have also identified key amino acid residues involved in proton binding, substrate recognition, and conformational transitions, providing valuable insights into the molecular determinants of transporter activity [11,39].

Collectively, these molecular features—including proton-coupled transport, broad substrate specificity, and structural adaptability—enable dipeptide transporters to function as versatile gateways for peptide and peptide-like molecules. These properties not only support physiological peptide transport but also create opportunities for exploiting peptide transport systems in therapeutic strategies, particularly in the context of transporter-mediated drug targeting [5,30,31].

## 3. Physiological Roles of Dipeptide Transport Systems

Dipeptide transport systems play essential roles in the absorption, distribution, and recycling of small peptides in mammalian physiology. Through the coordinated activity of peptide transporters belonging to the SLC15 family, cells can efficiently utilize peptide-derived nutrients and maintain systemic amino acid homeostasis. Among these transporters, PEPT1 and PEPT2 are responsible for the majority of dipeptide and tripeptide transport across plasma membranes in multiple tissues [3,5,7]. In this context, peptide transport can serve as a link between extracellular peptide pools generated through proteolysis and intracellular metabolic pathways (Figure 1).

One of the most well-characterized physiological functions of dipeptide transporters is their role in intestinal peptide absorption. In the small intestine, dietary proteins are initially degraded by gastric and pancreatic proteases into oligopeptides and free amino acids. Subsequent digestion by brush-border peptidases generates a mixture of dipeptides, tripeptides, and amino acids that are available for absorption by intestinal epithelial cells [3,41]. PEPT1, which is highly expressed on the apical membrane of enterocytes, mediates the uptake of these small peptides through proton-coupled transport [2,6,12]. Once transported into the cytoplasm, many dipeptides are rapidly hydrolyzed by intracellular peptidases into free amino acids, which can then enter systemic circulation and contribute to whole-body amino acid metabolism [3,5]. Importantly, peptide uptake via PEPT1 represents a highly efficient mechanism of nutrient absorption, as the transporter can accommodate a wide range of peptide substrates derived from dietary proteins.

In addition to intestinal absorption, peptide transporters also contribute to renal peptide reabsorption. During glomerular filtration, small peptides present in the bloodstream may enter the primary urine. To prevent excessive loss of valuable nitrogen sources, the kidney reabsorbs these peptides through transport systems located in the renal tubular epithelium [8,9]. PEPT2 is highly expressed in the apical membrane of proximal tubular cells and functions as a high-affinity transporter responsible for reclaiming filtered dipeptides and tripeptides from the tubular lumen [6,8]. Similar to intestinal peptide transport, reabsorbed peptides are typically hydrolyzed intracellularly into free amino acids, which can subsequently return to the systemic circulation.

Beyond the intestine and kidney, peptide transport systems are also expressed in a variety of extraintestinal tissues, including the lung, brain, and immune-related tissues, suggesting broader physiological functions [5,9,42]. In these tissues, peptide transporters may participate in local peptide recycling, nutrient sensing, and regulation of peptide-mediated signaling pathways. Members of the SLC15 family such as PHT1 and PHT2 have also been implicated in intracellular peptide transport within endosomal or lysosomal compartments, potentially contributing to antigen processing and immune responses [4,36]. Although the precise physiological roles of these transporters remain incompletely understood, their expression patterns indicate potential involvement in immune regulation and intracellular peptide trafficking.

Another important physiological aspect of peptide transport systems is their ability to mediate the cellular uptake of peptide-mimetic drugs. Because PEPT1 and PEPT2 recognize the general structural features of small peptides rather than specific amino acid sequences, a wide range of therapeutically relevant compounds can utilize these transporters for efficient cellular entry [5,12,30]. This property has been exploited in pharmacology to improve the oral bioavailability and tissue distribution of drugs that structurally resemble peptide substrates. For example, several β-lactam antibiotics, angiotensin-converting enzyme inhibitors, and antiviral prodrugs are known substrates of peptide transporters and rely on these systems for efficient absorption across epithelial barriers [13,14,15].

Taken together, these physiological roles highlight the importance of dipeptide transporters in nutrient assimilation, peptide recycling, and pharmacological transport processes. The ability of peptide transporters to handle structurally diverse substrates and operate across multiple tissues underscores their versatility in maintaining metabolic homeostasis. These fundamental physiological functions also provide the basis for exploring how peptide transport systems may be repurposed or dysregulated in pathological contexts, including cancer metabolism and therapeutic targeting strategies [31,33,43].

## 4. Expression of Peptide Transporters in Disease Contexts

Alterations in membrane transporter expression are a common feature of cancer cells and often reflect the metabolic adaptations required to sustain rapid proliferation and survival in nutrient-limited environments. In addition to the well-known upregulation of glucose and amino acid transporters, accumulating evidence suggests that dipeptide transport systems may also be altered in certain tumors, potentially contributing to metabolic flexibility and transporter-mediated uptake of peptide substrates [24,25,44]. In particular, members of the SLC15 transporter family, especially PEPT1 (SLC15A1) and PEPT2 (SLC15A2), have been reported to exhibit aberrant expression patterns in several cancer types.

Several studies have identified PEPT1 expression in gastrointestinal malignancies, including colon and gastric cancers (Table 2), where the transporter may be detected in tumor tissues or cancer-derived cell lines [24,26]. Because PEPT1 is normally expressed in intestinal epithelial cells, its presence in tumors of gastrointestinal origin may reflect both lineage-specific expression and tumor-associated metabolic demands. Functional analyses have suggested that cancer cells expressing PEPT1 are capable of actively transporting dipeptides and peptide-like compounds, indicating that the transporter remains functionally active in tumor cells [26,45].

In addition to gastrointestinal cancers, altered expression of peptide transporters has been reported in other malignancies. For example, PEPT1 expression has been observed in pancreatic ductal adenocarcinoma (PDAC) cells, where it may contribute to the uptake of peptide substrates and peptide-mimetic drugs [24,34]. In addition to expression studies, functional evidence has been reported showing that peptide transporter-mediated uptake can influence the pharmacokinetics and cellular delivery of peptide-modified anticancer agents, including amino acid ester prodrugs of gemcitabine [46]. Importantly, recent studies have further demonstrated that PEPT1 is not only expressed but functionally required for tumor growth. In PDAC models, inhibition of PEPT1 has been shown to significantly suppress cancer cell proliferation, supporting its role as a viable therapeutic target [47]. Interestingly, PEPT2 does not appear to compensate for PEPT1 function in this context. In PDAC cells, PEPT2 has been reported to exhibit aberrant subcellular localization, with predominant nuclear distribution rather than plasma membrane expression, suggesting that it is unlikely to contribute to functional peptide transport. This distinction may have important therapeutic implications, as selective inhibition of PEPT1 may not be offset by compensatory activity of PEPT2.

Similarly, hepatocellular carcinoma and cholangiocarcinoma have been reported to exhibit expression of peptide transport systems, although the physiological significance of this expression remains incompletely understood [24,25]. These observations raise the possibility that certain tumors may exploit peptide transport pathways to support nutrient acquisition or metabolic adaptation.

The expression of PEPT2 in cancer has been less extensively investigated, but several studies suggest that it may also be present in tumor tissues or cancer cell lines under specific conditions [4,42]. Given that PEPT2 is typically expressed in tissues such as the kidney, lung, and brain, its detection in tumors may reflect tissue-specific origins or adaptive responses to local metabolic conditions. However, the extent to which PEPT2 contributes to tumor metabolism or peptide uptake remains an area of ongoing investigation.

Recent advances in transcriptomic and proteomic analyses have further supported the idea that peptide transporter expression may be heterogeneous across tumor types. Large-scale cancer datasets have revealed variable expression patterns of SLC15 family members across different malignancies, suggesting that peptide transport capacity may differ substantially depending on tumor origin and microenvironmental conditions [25,48]. Such heterogeneity is consistent with the broader concept of metabolic plasticity in cancer, where tumor cells dynamically adjust transporter expression and nutrient acquisition strategies to adapt to fluctuating nutrient availability [17,18,19].

Another emerging consideration is the potential relationship between peptide transporter expression and the tumor microenvironment. Tumor tissues are characterized by high levels of extracellular proteolysis and protein degradation, processes that can generate small peptide fragments in the local microenvironment [49,50]. These peptide fragments may serve as potential substrates for dipeptide transporters expressed on cancer cells or stromal cells, thereby providing an additional nutrient source under metabolically stressed conditions. As illustrated in Figure 1, extracellular proteolysis may therefore generate peptide pools that could potentially interface with peptide transport systems and intracellular metabolism.

Overall, current evidence suggests that dipeptide transporters may be expressed in multiple cancer types and could contribute to tumor metabolic adaptation, nutrient uptake, or drug transport. However, the functional significance of peptide transporter expression in cancer remains incompletely understood, and further studies are required to clarify whether these transport systems play direct roles in tumor growth, metabolic reprogramming, or therapeutic responsiveness [24,25,44]. Understanding the regulation and functional consequences of peptide transporter expression in tumors will be essential for determining whether these transport systems represent viable targets for metabolic intervention or transporter-mediated drug delivery in cancer therapy.

**Table 2:** Reported expression or functional evidence of peptide transporters in selected cancer contexts. Several studies have reported the expression of peptide transporters in different malignancies. These observations suggest that peptide transport systems may contribute to tumor metabolism or facilitate transporter-mediated uptake of peptide-like drugs. However, the functional significance of peptide transporter expression in tumors remains incompletely understood and requires further investigation.

Cancer Type	Transporter Reported	Experimental System	Functional Implication	Therapeutic Relevance	Key References
Colon cancer	PEPT1	Human tumor tissues and colon cancer cell lines	Functional PEPT1 expression enables uptake of dipeptides and peptide-like substrates	Potential gateway for transporter-mediated delivery of peptide-mimetic drugs	[24,45]
Pancreatic ductal adenocarcinoma (PDAC)	PEPT1	PDAC cell lines	Functional requirement for tumor cell growth demonstrated in PDAC models	Validated therapeutic target with functional evidence	[35,47]
Hepatocellular carcinoma	PEPT1	Tumor tissue analyses and transporter expression studies	PEPT1 expression may support peptide-derived nutrient acquisition in tumor metabolism	Potential metabolic interface linking extracellular proteolysis and intracellular amino-acid pools	[24,25]
Cholangiocarcinoma	PEPT1	Hepatobiliary tumor cells and cancer models	Suggested involvement of peptide transport systems in uptake of peptide substrates	Possible transporter-dependent uptake of peptide-based drug conjugates	[24,25]
Lung cancer	PEPT2	Lung-derived cancer cell lines and expression studies	Presence of PEPT2-mediated peptide transport activity under specific cellular conditions	Possible contribution to uptake of peptide-mimetic compounds in lung tumors	[4,42]

## 5. Dipeptides as Potential Metabolic Intermediates in Tumor Microenvironments

Metabolic reprogramming is a hallmark of cancer and enables tumor cells to sustain rapid proliferation, survive under nutrient-limited conditions, and adapt to dynamic microenvironmental stresses. In addition to increased uptake of glucose and free amino acids, tumor cells frequently exploit alternative nutrient sources, including extracellular proteins and peptide fragments generated through proteolytic processes (Figure 1) [16,17,18,19]. To better contextualize the role of dipeptides within established metabolic frameworks, it is important to distinguish their contribution relative to canonical nutrient sources. Under most physiological conditions, free amino acids derived from direct transporter-mediated uptake or intracellular protein turnover represent the primary substrates fueling central metabolic pathways, including protein synthesis, nucleotide biosynthesis, and energy production [16,17,18,19].

Following transporter-mediated uptake via peptide transport systems such as PEPT1 and PEPT2, dipeptides are rapidly hydrolyzed by intracellular peptidases, thereby contributing to intracellular amino acid pools rather than functioning as independent metabolic entities [3,4,5,6]. In this context, dipeptides can be viewed as transient intermediates linking extracellular proteolysis to intracellular amino acid metabolism.

Cancer cells are known to engage multiple nutrient acquisition strategies beyond canonical glucose and amino acid uptake, including protein scavenging and macropinocytosis, which enable the utilization of extracellular proteins as alternative nutrient sources [20,21,22]. These processes are particularly relevant in tumor microenvironments characterized by extensive extracellular proteolysis mediated by enzymes such as matrix metalloproteinases, leading to the generation of peptide fragments of varying lengths [49,50].

In this setting, dipeptide uptake may represent an auxiliary and context-dependent nutrient source that complements established metabolic pathways. While their quantitative contribution is likely to be limited compared with canonical substrates under nutrient-replete conditions, peptide-derived intermediates may become more relevant under conditions of metabolic stress or nutrient limitation.

Recent advances in metabolomics and peptidomics have demonstrated the presence of circulating and tissue-associated dipeptides; however, the extent to which these peptide-derived nutrients contribute quantitatively to tumor metabolism remains incompletely defined [27,28,29]. More recently, high-throughput mass spectrometry-based and multi-omics approaches have enabled more comprehensive and spatially resolved profiling of metabolites and endogenous peptides in tumor tissues [51,52]. Future studies integrating metabolomic profiling with metabolic flux analysis will be required to clarify the relative contribution of dipeptide transport to cancer metabolism.

Dipeptides can arise from several sources within the tumor microenvironment. Extracellular proteins may undergo degradation through the activity of secreted proteases, matrix metalloproteinases, and other proteolytic enzymes, generating peptide fragments of varying lengths [49,50]. Proteolytic processing of extracellular proteins typically occurs in a stepwise manner. Initial cleavage is mediated by endopeptidases such as matrix metalloproteinases and cathepsins, which generate larger peptide fragments from extracellular matrix proteins and other substrates. These intermediate peptides are subsequently processed by exopeptidases, including aminopeptidases and dipeptidyl peptidases, which progressively trim peptides from their termini to produce shorter fragments, including dipeptides. In this context, dipeptides are unlikely to be direct products of primary proteolytic cleavage but rather arise from sequential processing of longer peptide intermediates. In addition, intracellular protein turnover mediated by proteasomal and lysosomal pathways can produce peptide intermediates that may enter extracellular spaces during cell damage or secretion processes [20,53]. These peptides may accumulate locally and become available for uptake through peptide transport systems expressed on tumor or stromal cells.

Although the biological significance of these observations remains unclear, altered levels of specific dipeptides in blood or tumor tissues suggest that peptide metabolism may be perturbed during tumor development.

Once transported into cells through peptide transporters such as PEPT1 or PEPT2, dipeptides are typically hydrolyzed by intracellular peptidases to generate free amino acids [3,5]. These amino acids can subsequently enter multiple metabolic pathways, including protein synthesis, nucleotide biosynthesis, and energy metabolism. In nutrient-limited tumor microenvironments, peptide-derived intermediates may therefore contribute to maintaining intracellular amino acid pools and supporting anabolic processes [23,54]. Protein scavenging pathways such as macropinocytosis may further expand this nutrient supply by enabling cancer cells to internalize extracellular proteins and degrade them into peptides and amino acids [21,22].

In addition to proteolytic activity, the tumor microenvironment is composed of diverse stromal and immune cell populations that actively contribute to extracellular protein turnover and peptide availability. Cancer-associated fibroblasts and other stromal cells play critical roles in extracellular matrix remodeling through the secretion of proteases, thereby influencing the generation and spatial distribution of peptide fragments within tumor tissues [49,50].

Immune cells infiltrating the tumor microenvironment may further contribute to local proteolytic processes and peptide dynamics. In particular, intracellular peptide transport systems such as members of the SLC15 family expressed in immune-related compartments have been implicated in peptide trafficking and immune signaling, suggesting potential roles in modulating peptide availability at the cellular and subcellular levels [36,42]. In this context, intracellular peptide transporters such as PHT1 (SLC15A4) and PHT2 (SLC15A3), which are expressed in immune-related compartments including endosomes and lysosomes, may also contribute to peptide handling within immune cells. These transporters have been implicated in peptide trafficking and immune signaling processes, raising the possibility that they could influence peptide availability and immune responses within the tumor microenvironment. However, their functional roles in cancer-associated immune contexts remain largely unexplored and require further investigation.

Moreover, the tumor microenvironment is inherently heterogeneous, with distinct regions exhibiting variations in oxygen availability, nutrient gradients, proteolytic activity, and cellular composition. As a result, peptide availability and utilization may differ across tumor regions, potentially influencing the functional relevance of peptide transport systems in a spatially dependent manner.

Despite increasing interest in peptide transport systems, several important questions remain unresolved. A key issue is the quantitative contribution of dipeptide-derived nutrients to tumor metabolism, which remains unclear in comparison with well-established nutrient sources such as glucose and free amino acids. In addition, although peptide transporters are expressed in various tumors, their functional significance is still uncertain. It is not yet clear whether transporter expression directly translates into meaningful metabolic flux or contributes to tumor growth under specific microenvironmental conditions. Another unresolved aspect concerns the relationship between peptide transport and intracellular metabolic signaling. Beyond serving as nutrient sources, it remains to be determined whether peptide-derived metabolites actively influence signaling networks. Finally, the translational potential of targeting peptide transport systems in cancer therapy remains uncertain. Despite promising preclinical findings, clinical validation is still limited, and key issues such as transporter specificity and regulation require further clarification.

Addressing these challenges will require integrative approaches combining metabolomics, peptidomics, and functional studies of transporter activity, which will be essential for defining the role of peptide transport systems in tumor biology and their potential as therapeutic targets.

## 6. Dipeptide Transporters as Gateways for Drug Delivery

Membrane transporters play a critical role in determining the cellular uptake, distribution, and pharmacokinetics of many therapeutic agents. Because dipeptide transporters exhibit broad substrate specificity and efficient uptake capacity, they have attracted considerable interest as potential targets for transporter-mediated drug delivery strategies. In particular, the ability of PEPT1 and PEPT2 to recognize and transport structurally diverse peptide-like molecules provides an opportunity to enhance drug absorption and intracellular delivery by designing compounds that mimic dipeptide structures [5,30,31,45]. Because peptide transporters recognize general peptide backbone structures rather than specific amino acid sequences, a wide range of peptide-mimetic drugs can potentially utilize these transport pathways for cellular entry.

The concept of exploiting peptide transport systems for drug delivery has been successfully demonstrated in several clinically approved drugs (Table 3). A notable example is valacyclovir, a prodrug of acyclovir in which the antiviral compound is conjugated to a valine moiety. This structural modification enables the drug to be recognized and transported by peptide transporters, significantly improving its intestinal absorption and oral bioavailability compared with acyclovir itself [14,15]. Similar strategies have been applied to other antiviral agents, antibiotics, and peptide-like drugs that rely on peptide transport systems for efficient uptake [13,45]. These examples highlight the feasibility of using peptide transporters as gateways for drug entry across epithelial barriers.

Despite these promising strategies, several limitations should be considered when evaluating their translational potential. A major challenge lies in the heterogeneous expression of peptide transporters such as PEPT1 and PEPT2, both across tumor types and within individual tumors, which may limit the reliability of transporter-targeted approaches. Moreover, the presence of these transporters in normal tissues, including the intestine and kidney, raises concerns regarding off-target uptake and potential toxicity, thereby reducing tumor specificity. Although peptide-modified drugs have shown encouraging results in preclinical studies, clinical validation remains limited, and most approaches have yet to progress beyond early development stages.

The pharmacological relevance of dipeptide transporters extends beyond intestinal absorption. Because PEPT1 and PEPT2 are expressed in multiple tissues, transporter-mediated uptake can influence the tissue distribution and intracellular accumulation of peptide-like drugs [43,44]. This property has prompted investigations into whether peptide transport systems could be exploited to selectively deliver therapeutic compounds to specific tissues or cell types that express these transporters. In principle, drugs designed to mimic dipeptide substrates could achieve enhanced uptake in cells expressing high levels of peptide transporters.

In the context of cancer, transporter-mediated drug delivery represents a potentially attractive therapeutic strategy. If tumor cells express functional dipeptide transporters, these transport systems could be harnessed to facilitate the selective uptake of peptide-modified anticancer agents or prodrugs [24,25]. Such an approach may improve intracellular drug concentrations in tumor cells while reducing systemic toxicity, particularly if transporter expression differs between malignant and normal tissues. Experimental studies have explored the use of dipeptide–drug conjugates, in which anticancer agents are chemically linked to dipeptide moieties to enhance transporter-mediated uptake [34,35]. Representative examples of dipeptide-based prodrug design further illustrate this concept. For instance, dipeptide conjugates such as Phe–Tyr–floxuridine and Ile–Gly–gemcitabine have been developed to exploit PEPT1-mediated uptake. In these constructs, anticancer agents are covalently linked to dipeptide moieties via ester or amide bonds, allowing them to mimic natural peptide substrates recognized by PEPT1. This design facilitates transporter-mediated cellular uptake and has been shown to enhance intracellular drug delivery in cancer cell models, including pancreatic cancer cells [34,35]. These conjugates are designed to be recognized by peptide transporters, transported into cells, and subsequently cleaved intracellularly to release the active drug.

Another potential strategy involves the development of transporter-targeted prodrugs, in which therapeutic compounds are chemically modified to resemble natural peptide substrates. Once transported into the cell, these prodrugs can be enzymatically converted into their active forms, thereby increasing intracellular drug availability [32,33,55]. Beyond their potential role as nutrient sources, certain dipeptides may also exert bioactive functions. Beyond their potential role as nutrient sources, certain dipeptides may also exert bioactive functions. Histidine-containing dipeptides such as carnosine and anserine have been reported to influence cellular metabolism, mitochondrial function, and redox balance [56,57,58]. However, it remains unclear whether these dipeptides are directly transported by SLC15 family members such as PEPT1 or PEPT2, and their relevance to peptide transporter-mediated uptake in cancer therefore remains speculative. This approach may be particularly useful for drugs with poor membrane permeability or limited bioavailability. By exploiting the natural transport capacity of peptide transporters, transporter-targeted prodrugs can overcome barriers associated with passive diffusion or inefficient uptake.

Despite these promising concepts, several challenges must be addressed before peptide transporter-targeted drug delivery can be fully realized in cancer therapy. One major consideration is the heterogeneous expression of peptide transporters across different tumor types and tissues, which may limit the universality of transporter-targeted strategies [25,48]. In addition, the broad substrate specificity of peptide transporters means that transporter-mediated uptake may also occur in normal tissues, potentially affecting drug distribution and toxicity profiles [43,44]. A detailed understanding of transporter expression patterns and functional activity in tumors will therefore be essential for optimizing therapeutic selectivity.

Nevertheless, the unique combination of broad substrate recognition, efficient transport capacity, and pharmacological accessibility makes dipeptide transporters attractive candidates for drug targeting strategies. Continued research into the structural and functional properties of these transport systems, together with advances in medicinal chemistry and prodrug design, may enable the development of new transporter-mediated therapeutic approaches that exploit peptide transport pathways for improved cancer treatment [31,33,43].

## 7. Emerging Therapeutic Strategies Exploiting Dipeptide Transport Systems

Growing recognition of the functional roles of peptide transporters in cellular metabolism and drug uptake has stimulated interest in developing therapeutic strategies that exploit dipeptide transport systems as pharmacological targets. Although research in this area remains at an early stage, several emerging approaches have been proposed to utilize peptide transport pathways for improving anticancer drug delivery, enhancing therapeutic selectivity, or modulating tumor metabolism [24,25,43]. In principle, peptide transporters may represent a functional interface between extracellular peptide pools and intracellular metabolic pathways, creating opportunities for transporter-mediated therapeutic intervention.

One promising strategy involves the development of dipeptide–drug conjugates designed to exploit transporter-mediated uptake. In this approach, anticancer agents are chemically linked to dipeptide moieties that can be recognized by peptide transporters such as PEPT1 or PEPT2. Once transported into the cell, intracellular enzymes can cleave the conjugate to release the active drug molecule [34,35]. This strategy may enhance intracellular drug accumulation in transporter-expressing cells while improving drug solubility and pharmacokinetic properties. Several experimental studies have explored peptide-conjugated forms of anticancer compounds with the aim of improving transporter-mediated uptake and tumor targeting [34,35].

Another approach focuses on the design of transporter-targeted prodrugs. In contrast to direct drug conjugates, prodrugs are inactive or weakly active compounds that are metabolically converted into active drugs after cellular uptake. By incorporating structural elements that mimic dipeptide substrates, prodrugs can be engineered to utilize peptide transport systems for efficient cellular entry [32,33,55]. Following transporter-mediated uptake, intracellular enzymes such as esterases or peptidases convert the prodrug into its active form. This strategy has already proven effective in improving the oral bioavailability of several drugs and may be adaptable for cancer therapeutics [31,33].

To date, direct pharmacological inhibition of peptide transporters has rarely been explored in cancer therapy, and most existing studies have instead focused on exploiting transporter activity for drug delivery. Whether selective inhibition of peptide transport pathways could sensitize tumors to conventional anticancer therapies remains an open and largely unexplored question.

In addition to enhancing drug delivery, peptide transport systems may also be considered as targets for metabolic intervention. If tumor cells rely on dipeptide uptake to support metabolic demands under nutrient-limited conditions, inhibition of peptide transport could theoretically disrupt tumor nutrient acquisition pathways [23,54]. Although this concept remains largely hypothetical, pharmacological inhibition of peptide transporters could potentially alter intracellular amino acid availability or interfere with peptide-dependent metabolic processes in cancer cells.

Another emerging concept involves the use of peptide transport systems to facilitate targeted delivery of imaging agents or diagnostic probes. Because membrane transporters can influence the intracellular accumulation of small molecules, transporter-targeted probes could potentially be used to visualize transporter activity in tumors using molecular imaging techniques [43,44]. Such approaches may help identify tumors that exhibit elevated peptide transporter expression and could therefore be suitable candidates for transporter-targeted therapeutic strategies.

Despite these potential applications, several challenges remain for the development of transporter-based therapeutic strategies. One important issue is the heterogeneity of transporter expression among different tumor types and even within individual tumors [25,48]. This variability may complicate the prediction of therapeutic responses to transporter-targeted drugs. In addition, peptide transporters are expressed in multiple normal tissues, including the intestine and kidney, which may influence systemic drug distribution and raise concerns about off-target effects [43,44].

Because peptide transport systems naturally mediate the cellular uptake of small peptides, they represent an attractive molecular entry point for designing transporter-directed therapeutic strategies. Future progress in this field will likely depend on improved characterization of transporter expression patterns in cancer, better understanding of transporter substrate recognition mechanisms, and advances in medicinal chemistry that enable the design of optimized transporter substrates. Integrating knowledge from cancer metabolism, membrane transporter biology, and drug design may ultimately allow peptide transport systems to be harnessed as effective tools for transporter-mediated targeting of anticancer therapies [24,43,45].

## 8. Challenges and Future Perspectives

Despite growing interest in the potential roles of dipeptide transporters in cancer biology and therapeutic targeting, several important challenges remain before these transport systems can be fully exploited for clinical applications. A deeper understanding of the regulation, functional significance, and tissue-specific expression of peptide transporters will be essential for determining whether they represent viable targets for metabolic intervention or transporter-mediated drug delivery strategies in oncology [5,31,43,59]. As illustrated in Figure 1, peptide transport systems may represent an interface linking extracellular proteolysis-derived peptide pools with intracellular metabolic pathways.

One major challenge is the limited understanding of the functional role of dipeptide transporters in tumor metabolism. Although altered expression of peptide transporters has been reported in several cancer types, it remains unclear whether this expression directly contributes to tumor growth or represents a secondary consequence of cellular transformation [24,25,59]. Unlike glucose and amino acid transporters, whose metabolic roles in cancer are well established, the contribution of peptide-derived nutrients to tumor metabolic networks has received comparatively little attention [59,60,61]. Determining whether cancer cells actively utilize dipeptides as metabolic substrates and how peptide uptake integrates with broader metabolic pathways will be critical for clarifying the biological significance of peptide transport in tumors.

Another important consideration is the heterogeneous expression of peptide transporters across tissues and tumor types. While certain cancers appear to express PEPT1 or PEPT2, expression levels may vary widely depending on tumor origin, differentiation status, and microenvironmental conditions [25,48]. In addition, peptide transporters are naturally expressed in several normal tissues, including the small intestine, kidney, and lung [3,5,59]. This widespread physiological expression may complicate efforts to achieve tumor-specific targeting using transporter-mediated drug delivery strategies, as drugs designed to exploit peptide transporters may also be taken up by normal tissues.

The substrate promiscuity of peptide transporters, although advantageous for nutrient absorption and drug delivery, also presents challenges for therapeutic design. Because PEPT1 and PEPT2 recognize a broad range of peptide-like structures, predicting transporter specificity and optimizing drug–transporter interactions can be complex [10,11,12]. Structural studies of SLC15 transporters have begun to provide insights into the molecular basis of substrate recognition and prodrug transport [62]. These advances may facilitate the rational design of transporter-targeted therapeutics and peptide-based drug delivery systems [63,64].

Another area requiring further investigation is the potential role of peptide transporters in the tumor microenvironment. Tumor tissues are characterized by extensive extracellular matrix remodeling and proteolysis, processes that can generate peptide fragments within the local environment [49,50]. Whether these peptides are actively utilized by tumor cells through peptide transport systems remains largely unexplored. Understanding how extracellular peptide pools are generated, transported, and metabolized within tumor ecosystems may provide new insights into tumor nutrient acquisition strategies and reveal previously unrecognized metabolic interactions between cancer cells and stromal components.

Future studies integrating metabolomics, peptidomics, and transcriptomic analyses may help clarify the role of dipeptides and peptide transport systems in cancer. High-throughput profiling approaches could identify tumor types that exhibit elevated peptide transporter expression or altered peptide metabolite signatures [65]. In addition, improved experimental models—including transporter knockdown systems, genetically engineered mouse models, and transporter-specific imaging probes—may enable more precise evaluation of the functional roles of peptide transporters in tumor biology and therapeutic targeting [43,44].

Despite more than two decades of research on peptide transporter-targeted drug delivery, no PEPT1-directed therapies have yet advanced to clinical trials. Several factors may contribute to this translational gap. One major challenge lies in optimizing dosing strategies and pharmacokinetics, as transporter-mediated uptake can be influenced by substrate competition, saturation effects, and variable expression levels across tissues.

In addition, PEPT1 is physiologically expressed in normal tissues such as the intestine, raising concerns regarding on-target but off-tumor toxicity, which may limit the therapeutic window of transporter-targeted approaches. Furthermore, demonstrating clear clinical benefit over established treatment modalities remains challenging, particularly given the complexity of tumor metabolism and the availability of effective standard therapies.

Addressing these barriers will require improved strategies for achieving tumor selectivity, a better understanding of transporter regulation in disease contexts, and rigorous evaluation in clinically relevant models.

In conclusion, dipeptide transport systems represent an intriguing but still incompletely understood interface between metabolism, membrane transport, and therapeutic targeting in cancer. Importantly, the emerging recognition of peptide metabolites within tumor microenvironments suggests that small peptides may represent an additional layer of metabolic complexity in cancer biology. While current evidence indicates that peptide transporters may contribute to tumor metabolic adaptation and drug uptake, their precise biological roles remain incompletely understood. Continued research into the regulation and function of peptide transporters, together with advances in drug design and metabolic profiling, may ultimately reveal new opportunities for exploiting peptide transport pathways in cancer diagnosis and therapy.

## Figures and Tables

**Table 1:** Members of the SLC15 peptide transporter family and their physiological characteristics. The SLC15 family consists of four proton-coupled oligopeptide transporters that mediate the transport of dipeptides, tripeptides, and peptide-like molecules across cellular membranes. PEPT1 and PEPT2 primarily function in nutrient absorption and peptide reabsorption, whereas PHT1 and PHT2 are involved in intracellular peptide transport and immune signaling.

Transporter	Gene Name	Major Tissue Distribution	Transport Characteristics	Physiological Role	Key References
PEPT1	*SLC15A1*	Small intestine, gastrointestinal epithelium	Low-affinity, high-capacity proton-coupled transporter	Absorption of dietary dipeptides and tripeptides	[1,6]
PEPT2	*SLC15A2*	Kidney, lung, brain, mammary gland	High-affinity, low-capacity proton-coupled transporter	Reabsorption of filtered peptides and peptide homeostasis	[3,5]
PHT1	*SLC15A4*	Immune cells, lysosomes, endosomes	Intracellular peptide transport	Immune signaling and peptide trafficking	[36]
PHT2	*SLC15A3*	Immune-related tissues	Intracellular peptide transport	Lysosomal peptide transport and immune regulation	[36]

**Table 3:** Representative therapeutic agents that utilize peptide transport systems for absorption or cellular uptake. Several clinically used drugs structurally resemble dipeptides or peptide-like molecules and can utilize peptide transporters, particularly PEPT1 and PEPT2, for efficient intestinal absorption or cellular uptake. Exploiting peptide transport systems has therefore become an important strategy in prodrug design aimed at improving drug bioavailability and pharmacokinetic properties.

Drug	Drug Class	Therapeutic Indication	Transporter Involvement	Notes	Key References
Valacyclovir	Antiviral prodrug	Herpes virus infections	PEPT1	Improved oral bioavailability compared with acyclovir	[14,55]
Valganciclovir	Antiviral prodrug	Cytomegalovirus infections	PEPT1	Peptide-like prodrug enabling efficient absorption	[33,55]
β-lactam antibiotics	Antibiotics	Bacterial infections	PEPT1/PEPT2	Structural similarity to dipeptides enables transporter-mediated uptake	[6,12]
ACE inhibitors (e.g., captopril derivatives)	Antihypertensive agents	Cardiovascular diseases	PEPT1	Peptide-like structure facilitates intestinal absorption	[5,55]
Amino acid ester prodrugs	Transporter-targeted prodrug design	Various	PEPT1	Rational prodrug strategy to enhance intestinal absorption	[33]

## Data Availability

No new data were created or analyzed in this study. Data sharing is not applicable to this article.

## Abbreviations

The following abbreviations are used in this manuscript:

ACE	Angiotensin-Converting Enzyme
ECM	Extracellular Matrix
MFS	Major Facilitator Superfamily
PDAC	Pancreatic Ductal Adenocarcinoma
PEPT1	Peptide Transporter 1
PEPT2	Peptide Transporter 2
PHT1	Peptide/Histidine Transporter 1
PHT2	Peptide/Histidine Transporter 2
SLC	Solute Carrier
SLC15	Solute Carrier Family 15
SLC15A1	Solute Carrier Family 15 Member 1
SLC15A2	Solute Carrier Family 15 Member 2
SLC15A3	Solute Carrier Family 15 Member 3
TME	Tumor Microenvironment
